# Low HALP Score Predicts Prolonged Hospitalization in Solid Tumor Patients with Febrile Neutropenia

**DOI:** 10.3390/curroncol33010014

**Published:** 2025-12-27

**Authors:** Salih Karatlı, Doğan Yazılıtaş

**Affiliations:** Department of Medical Oncology, Etlik City Hospital, 06170 Ankara, Türkiye

**Keywords:** HALP, PNI, CAR, CISNE, febrile neutropenia, length of hospital stay, solid tumor

## Abstract

Background: Febrile neutropenia (FN) is a serious chemotherapy-related complication in patients with solid tumors. Identifying simple and accessible biomarkers that can predict prolonged hospitalization may support early risk stratification and clinical decision-making. Methods: This retrospective study included 169 adults hospitalized with FN between January 2023 and January 2025. Immunonutritional indices, including the Hemoglobin-Albumin-Lymphocyte-Platelet (HALP) score, the Prognostic Nutritional Index (PNI), and the C-reactive protein/albumin ratio (CAR), as well as the Clinical Index of Stable Febrile Neutropenia (CISNE) score were calculated. HALP and PNI were categorized using ROC-derived cut-offs based on the Youden Index. Prolonged hospital stay was defined as a binary variable based on the cohort median (>9 days). Spearman correlation, univariate and multivariate logistic regression were performed to identify predictors of prolonged hospitalization. Results: HALP showed a significant negative correlation with hospitalization duration (r = −0.469; *p* < 0.001), as did serum albumin (r = −0.184; *p* = 0.017) and PNI (r = −0.273; *p* < 0.001). CAR (*p* = 0.617) and neutrophil count (*p* = 0.955) demonstrated no correlation. In univariate logistic regression, low HALP (*p* < 0.001), low PNI (*p* = 0.001), intermediate CISNE (*p* = 0.002), high CISNE (*p* < 0.001), microbiological culture positivity (*p* < 0.001), and sex (*p* = 0.015) were significantly associated with prolonged hospitalization. Age, comorbidity status, metastatic stage, and CAR were not significant. In the multivariate model, low HALP (*p* < 0.001), intermediate CISNE (*p* = 0.007), high CISNE (*p* < 0.001), and culture positivity (*p* < 0.001) remained independent predictors. PNI (*p* = 0.400) and sex (*p* = 0.176) did not retain significance. Conclusions: A Low HALP score, higher CISNE risk categories, and microbiological culture positivity independently predicted prolonged hospitalization in FN. HALP, as a simple and inexpensive immunonutritional marker, may enhance early FN risk assessment when used alongside validated clinical tools such as CISNE or MASCC.

## Simple Summary

Febrile neutropenia (FN) is a serious complication of chemotherapy that often requires hospitalization. Identifying simple and accessible markers that can help predict clinical outcomes is important for improving patient management. In this retrospective study, we evaluated the relationship between the Hemoglobin–Albumin–Lymphocyte–Platelet (HALP) score, the C-reactive protein/albumin ratio (CAR), and the Prognostic Nutritional Index (PNI) with the length of hospital stay in 169 solid tumor patients hospitalized with FN. Our findings showed that a low HALP score, intermediate and high Clinical Index of Stable Febrile Neutropenia (CISNE) risk categories, and positive microbiological culture were independent predictors of prolonged hospitalization. In contrast, CAR showed no meaningful association, and PNI lost significance after adjustment for other clinical factors. These results suggest that HALP, together with clinical risk tools such as CISNE, may serve as a practical and low-cost marker to support early risk stratification in FN.

## 1. Introduction

Febrile neutropenia (FN) is defined in accordance with internationally accepted guidelines of the American Society of Clinical Oncology (ASCO), the Infectious Diseases Society of America (IDSA), and the European Society for Medical Oncology (ESMO) as the presence of a single oral temperature measurement ≥38.3 °C or a sustained temperature ≥ 38.0 °C lasting longer than one hour, in conjunction with an absolute neutrophil count (ANC) < 500 cells/mm^3^, or an ANC between 500 and 1000 cells/mm^3^ with an anticipated decline to <500 cells/mm^3^ within the subsequent 48 h. FN is an oncologic emergency that can be life-threatening and significantly increases the risk of serious infections [1]. It is one of the most common complications that can develop during chemotherapy treatment, occurring in more than 80% of patients with hematologic malignancies and in approximately 50% of those with solid tumors [2]. FN increases the risk of sepsis and septic shock, leading to prolonged hospital stays, delays in chemotherapy cycles, and increased overall mortality rates. It has been reported that sepsis develops in approximately half of the patients, while septic shock occurs in about 10%. The overall mortality rate ranges between 5 and 20%, and this rate may reach up to 50% in cases of shock [3,4]. In particular, a decrease in absolute neutrophil count (ANC) below 100 cells/mm^3^ is considered profound neutropenia, and in this condition, the risk of severe infection increases significantly. Therefore, the early recognition of FN, accurate risk stratification, and timely implementation of appropriate treatment strategies are of great importance not only for oncology clinicians but also for physicians in primary care, emergency departments, internal medicine, and intensive care units. Since FN management directly affects hospital stay duration, complication rates, and chemotherapy continuity, close adherence to current guidelines is recommended in the evaluation and follow-up of these patients. The ability to predict the clinical course and possible complication risks of these patients during hospitalization is extremely important for planning early intervention strategies and improving patient prognosis [2,5].

In the evaluation of cancer prognosis, biochemical parameters reflecting the patient’s nutritional status and systemic inflammatory response have emerged as important prognostic markers in recent years. In this context, the HALP score (Hemoglobin–Albumin–Lymphocyte–Platelet), CAR score (C-reactive protein (CRP)/Albumin ratio) and, PNI (Prognostic Nutritional Index) are considered composite indicators that reflect both nutritional status and inflammatory activity. In addition, the inflammation-based Systemic Immune-Inflammation Index (SII) and Pan-Immune-Inflammation Value (PIV) are among other important parameters whose prognostic value has been demonstrated in various malignancies. Although the effects of these scores on tumor progression, treatment response, and overall survival have been extensively investigated in different cancer types, their relationships with the clinical course in oncology patients who develop FN have not been sufficiently elucidated [6,7,8,9,10,11].

During FN, acute systemic inflammation, cytokine release, capillary leakage, and catabolic stress lead to rapid declines in albumin levels, consumption of lymphocytes and platelets, and reductions in hemoglobin. These alterations reflect impaired immune competence, reduced tissue oxygenation, and a more severe infectious burden. Because the HALP score integrates these four parameters into a single composite indicator, low HALP values biologically correspond to heightened inflammatory activity, profound immunonutritional deterioration, and consequently, a greater likelihood of prolonged hospitalization.

One of the most important tools developed for risk stratification of FN is the Clinical Index of Stable Febrile Neutropenia (CISNE) score. This score, which includes 6 parameters—ECOG performance status, chronic obstructive pulmonary disease, chronic cardiovascular disease, mucositis, albumin level, and stress hyperglycemia—has been found to be superior to MASCC in predicting serious complications, particularly in patients with FN who appear clinically stable. However, the number of studies examining the relationship between the CISNE score and length of hospital stay remains very limited [12,13].

In this study, it was aimed to evaluate the relationship between HALP, CAR, and PNI scores and the length of hospital stay in patients who developed FN. In addition to these immunonutritional markers, the CISNE score was also assessed as a validated clinical risk stratification tool, given its potential relevance to hospitalization outcomes. The evaluation of these parameters may contribute to the identification of simple, accessible, and low-cost biomarkers that can guide early risk stratification and prediction of the clinical course in FN patients.

## 2. Materials and Methods 

### 2.1. Patients

The study was conducted at Etlik City Hospital, a large tertiary-care referral center located in Ankara, Türkiye. The hospital includes a comprehensive oncology department and evaluates approximately 9000–10,000 oncology outpatient visits annually, serving a metropolitan population of nearly six million.

Ethical approval for this retrospective study was obtained from the Etlik City Hospital Clinical Research Ethics Committee on 22 October 2025 (Ethics Committee No: 2025-551), prior to data extraction and analysis. The dataset consisted of adult patients with solid tumors who had been hospitalized in the Medical Oncology Service of Etlik City Hospital due to FN between 1 January 2023, and 1 January 2025. A total of 178 patient records were screened: nine with missing laboratory data were excluded, and data from 169 patients were included in the final analysis.

#### 2.1.1. Inclusion Criteria

- Aged 18 years or older;
- Diagnosed with a solid tumor;
- Hospitalized due to FN;
- Complete pre-treatment laboratory data available.

#### 2.1.2. Exclusion Criteria

- Diagnosis of hematologic malignancy;
- Missing or inaccurate laboratory data.

### 2.2. Data Collection and Laboratory Parameters

Patient data, including age, sex, primary tumor type, disease stage, presence of comorbidities, microbiological culture results, and length of hospital stay, were retrospectively obtained from the hospital information management system. Laboratory parameters included hemoglobin (g/dL), albumin (g/L), lymphocyte (/µL), Monocyte (/µL), platelet (/µL), and CRP (mg/L) levels. All laboratory parameters, including neutrophil count and albumin, were obtained at admission prior to therapeutic interventions. Blood cultures were routinely obtained from all patients at admission, whereas urine, respiratory, and stool cultures were requested selectively according to the clinical presentation.

FN management was performed in accordance with our institutional protocol and international guidelines. All patients received empiric broad-spectrum antibiotic therapy at the time of diagnosis, and granulocyte colony-stimulating factor (G-CSF) support was provided. G-CSF treatment was continued until neutrophil recovery and clinical stabilization were achieved.

The following immunonutritional and inflammation-based indices were calculated:

HALP score = Hemoglobin (g/dL) × Albumin (g/L) × Lymphocyte (/µL) ÷ Platelet (/µL)
CAR = CRP (mg/L)/Albumin (g/L)
PNI = 10 × Serum Albumin (g/dL) + 0.005 × Lymphocyte (/µL).

Albumin values were recorded in g/L for routine laboratory reporting and were used in this unit for the calculation of the HALP score and CAR, in accordance with their original definitions. For the calculation of the PNI, albumin values were converted to g/dL, as required by the original PNI formula.

Additionally, CISNE score was calculated based on six parameters using clinical information obtained from patient discharge summaries and laboratory data recorded in the hospital electronic medical system:

- ECOG performance status ≥ 2 (2 points);
- Chronic obstructive pulmonary disease (1 point);
- Chronic cardiovascular disease (1 point);
- NCI (National Cancer Institute) mucositis grade ≥ 2 (1 point);
- Albumin ≤ 3.5 g/dL (1 point);
- Stress hyperglycemia (glucose > 121 mg/dL without diabetes or >250 mg/dL with diabetes) (2 points).

Total score ranges from 0 to 8 points. Patients were stratified as low risk (0 points), intermediate risk (1–2 points), and high risk (≥3 points).

### 2.3. Statistical Analysis

Statistical analyses were performed using IBM SPSS Statistics, version 25.0 (IBM Corp., Armonk, NY, USA). Median (minimum–maximum) values were used for continuous variables, and frequency (*n*, %) for categorical variables.

The relationships between immunonutritional indices (HALP, PNI, CAR) and length of hospital stay were assessed using Spearman’s correlation test.

Receiver operating characteristic (ROC) curve analysis was used to determine optimal cut-off values for HALP and PNI. Optimal thresholds were identified based on the Youden Index (J = Sensitivity + Specificity − 1), which maximizes overall diagnostic performance. Based on these cut-offs, both indices were categorized into low and high groups.

Prolonged hospital stay was analyzed as a binary outcome based on the median length of stay, which served as the cut-off point. Prolonged hospitalization was operationally defined as a hospital stay longer than the cohort median (9 days). This binary outcome (≤9 days vs. >9 days) was used for both ROC analysis and logistic regression models. Throughout the manuscript, the term “prolonged hospitalization” refers to a hospital stay longer than the median duration (>9 days). Age was categorized into two groups (<median vs. ≥median) for use in univariate and multivariate logistic regression models.

Univariate logistic regression analysis was performed to identify factors associated with prolonged hospitalization. Variables with *p* < 0.05 in univariate analysis were included in the multivariate model to determine independent predictors. A *p*-value < 0.05 was considered statistically significant. Multicollinearity was assessed using Variance Inflation Factor (VIF) values for all variables included in the multivariate logistic regression model.

## 3. Results 

A total of 169 oncology patients hospitalized due to FN were included in the study. The median age of the patients was 64 years (19–81), and 50.9% were female. The most common primary diagnoses were lung (31.4%), head and neck (16.0%), and breast (14.2%) cancers. More than half of the cohort (51.8%) had metastatic disease, and 50.9% had at least one comorbid condition. Growth in microbiological cultures was detected in 33.7% of patients. According to the CISNE score distribution, 30.2% of patients were classified as low risk, 39.6% as intermediate risk, and 30.2% as high risk. The median length of hospital stay was 9 days (3–29) (Table 1).

When laboratory findings were examined, the median hemoglobin level was 9.6 g/dL, albumin 32 g/L, and CRP 99 mg/L. The median neutrophil count was 120/µL (range, 10–940), and the median lymphocyte count was 370/µL (range, 10–1860). The median HALP score was 17.46 (range, 0.8–276), the median CAR score was 3.33 (range, 0.62–22.22), and the median PNI score was 35.50 (range, 13.35–60.30) (Table 2).

Microbiological culture growth was detected in 57 patients (33.7%). Positive cultures included urine, blood, respiratory, and stool specimens, with some patients showing positivity at multiple sites. A summary of microbiological culture results is presented in Table 3.

According to the Spearman correlation analysis, a significant negative correlation was observed between the HALP score and the length of hospital stay (r = −0.469, *p* < 0.001). A significant negative correlation was also found for serum albumin levels (r = −0.184, *p* = 0.017) and for the PNI score (r = −0.273, *p* < 0.001). In contrast, no significant relationship was identified for the CAR score (r = −0.039, *p* = 0.617) or neutrophil count (r = 0.004, *p* = 0.955) (Table 4).

**Figure 1:**
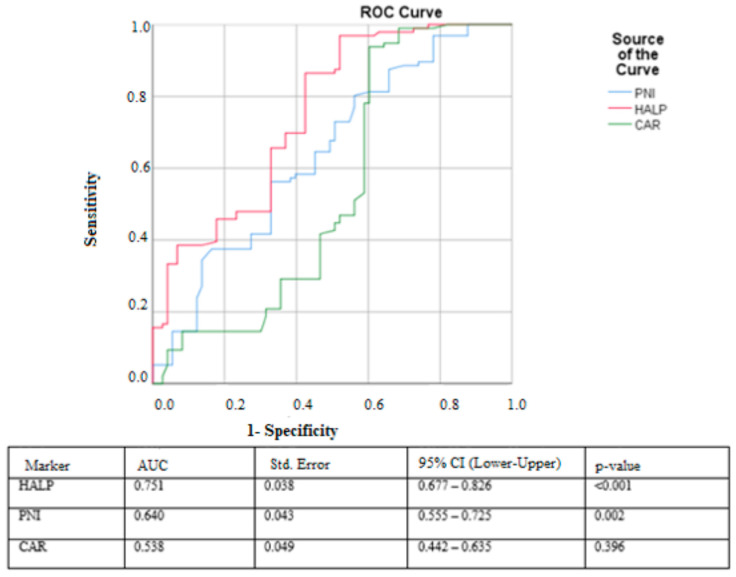
ROC curves of HALP, PNI, and CAR for predicting prolonged hospitalization in patients with febrile neutropenia.

In the ROC curve analysis, the HALP score demonstrated the strongest discriminatory performance for predicting prolonged hospital stay, with an AUC of 0.751 (95% CI: 0.677–0.826, *p* < 0.001). The optimal cut-off value for HALP was 5.83, yielding a sensitivity of 96.9% and a specificity of 52.1%. The PNI score showed moderate predictive ability, with an AUC of 0.640 (95% CI: 0.555–0.725, *p* = 0.002), and a cut-off value of 30.15 provided 80.2% sensitivity and 56.2% specificity. In contrast, the CAR score exhibited poor discriminatory performance, with an AUC of 0.538 (95% CI: 0.442–0.635, *p* = 0.396); its cut-off value of 1.30 produced 93% sensitivity but only 60% specificity (Figure 1).

In the univariate logistic regression analysis, several variables were significantly associated with prolonged hospital stay, including low HALP score (OR = 28.57; *p* < 0.001), low PNI score (OR = 3.16; *p* = 0.001), intermediate CISNE risk category (OR = 4.756; *p* = 0.002), high CISNE risk category (OR = 30.750; *p* < 0.001), microbiological culture positivity (OR = 8.395; *p* < 0.001), and sex (OR = 0.464; *p* = 0.015). In contrast, metastatic disease status (*p* = 0.107), age (*p* = 0.084), and comorbidity status (*p* = 0.551) were not significantly associated with prolonged hospitalization in univariate analysis. CAR was not included in the regression models because it did not show a significant association with hospitalization duration in either correlation or ROC analysis.

Variables with *p* < 0.05 in the univariate analysis were subsequently included in a multivariate logistic regression model to identify independent predictors of prolonged hospital stay. In the adjusted model, low HALP score (OR = 47.62; *p* < 0.001), intermediate CISNE risk category (OR = 8.620; *p* = 0.007), high CISNE risk category (OR = 67.49; *p* < 0.001), and culture positivity (OR = 11.279; *p* < 0.001) remained independent predictors, whereas PNI lost its statistical significance after adjustment (*p* = 0.400) and sex, despite being significant in the univariate model, also did not retain significance in the multivariate analysis (*p* = 0.176) (Table 5). In the multicollinearity analysis, all variables demonstrated VIF values below 2.5, indicating that no meaningful multicollinearity was present in the model.

## 4. Discussion 

This study demonstrated that the HALP score has a significant predictive value for the length of hospital stay in patients with solid tumors who were hospitalized due to FN. Both univariate and multivariate analyses confirmed that a low HALP score was one of the strongest independent predictors of prolonged hospitalization. In addition, the CISNE risk score—particularly the intermediate and high-risk categories—emerged as an important independent determinant of hospital stay, underscoring the clinical value of this composite scoring system in FN assessment.

The HALP score has been shown to have prognostic and predictive value across various malignancies, and the cut-off values reported in the literature vary considerably among different cancer types. In our study, the HALP cut-off value was determined as 5.83. Compared with the cut-off values reported in non-FN oncology populations (typically 15–40), the markedly lower threshold observed in this cohort may reflect the acute and profound immunonutritional deterioration characteristic of FN. This finding suggests that FN represents a biologically distinct clinical condition and that the HALP score may serve as a more sensitive early risk indicator in this specific patient population.

FN is a serious chemotherapy-related complication and a major cause of morbidity and hospitalization in patients with solid tumors. In the literature, the average length of hospital stay due to FN typically ranges from 6 to 14 days, with previous studies reporting mean durations of approximately 10 days in adults, 6–8 days in older patients, and around 8 days in pediatric cases [5]. In a large cohort of 5809 adult cancer patients, Chindaprasirt et al. (2013) reported a median hospital stay of 8.67 days [14], while Bachlitzanaki et al. (2023) found a median of 9 days (range 3–43) in solid tumor patients [15]. In our study, the hospitalization duration of solid tumor patients with FN was consistent with these findings, indicating that FN generally necessitates inpatient management lasting more than one week.

The literature indicates that pneumonia, bacteremia, sepsis, hypotension, invasive fungal infections, low MASCC scores, and prolonged neutropenia are key factors associated with extended hospitalization and poor prognosis in FN cases. Large series further show that mortality is markedly higher in patients who develop pneumonia or sepsis, and that prolonged FN-related hospital stays are frequently accompanied by low MASCC scores, thrombocytopenia, and elevated procalcitonin, IL-6, and D-dimer levels [16,17,18].

In our study, since no mortality was observed among the patients monitored in the ward and the number of patients transferred to the intensive care unit was insufficient for statistical analysis, the length of hospital stay was used as an indirect clinical endpoint. No significant association was found between the length of hospital stay and comorbidity status, age, sex, or neutrophil count. In contrast, patients with positive microbiological cultures had a significantly longer hospitalization compared with culture-negative patients. These findings are partially consistent with the literature, although the lack of an effect of comorbidities and neutrophil count is notable. Standardized FN management protocols, including broad-spectrum antibiotics and G-CSF, may rapidly correct neutropenia and reduce the clinical impact of comorbidities, thereby minimizing variation in hospitalization duration. Moreover, most comorbidities in our cohort were mild or well controlled, which may further explain the absence of association. Future studies using more sensitive measures of comorbidity burden, such as the Charlson Comorbidity Index, may provide clearer insights.

Recent research has increasingly focused on identifying prognostic indicators that can predict not only the development of FN but also mortality and clinical deterioration. Several nutrition- and inflammation-based biomarkers have been evaluated in this context. Serum albumin is one of the most notable markers, as it reflects both systemic inflammation and nutritional status. In a prospective study by Dimitrijević et al. (2024), low albumin levels were strongly associated with FN-related and 28-day mortality, and adding albumin to the MASCC index improved predictive accuracy by 50–66% [19]. Similarly, the Mean Platelet Volume-to-Albumin Ratio (MPV/ALB) was identified as a robust predictor of early FN-related mortality [20]. Other indices—such as the Geriatric Nutritional Risk Index (GNRI), the Combined Platelet-Neutrophil/Lymphocyte Ratio (COP-NLR), and the Modified Glasgow Prognostic Score (mGPS)—have also been reported to independently predict FN risk prior to chemotherapy [21].

In our study, the lack of an association between the CAR score and length of hospital stay may be attributed to the rapid decline of CRP levels during the early phase of FN treatment and the limited short-term variability of serum albumin. The loss of significance of the PNI score in the multivariate model can be explained by the fact that PNI incorporates only two parameters (albumin and lymphocyte count) and therefore provides more limited biological information compared with composite indices such as the HALP score, which more comprehensively represent inflammation, immune response, and hematologic status. HALP includes hemoglobin and platelet values in addition to albumin and lymphocytes, offering a broader immunonutritional profile; consequently, in a multifactorial condition such as FN, HALP demonstrated stronger predictive power than PNI. Thus, when HALP is included in the model, the independent prognostic contribution of PNI diminishes.

The CISNE score is a validated and reliable risk stratification tool for predicting serious complications and early adverse outcomes in clinically stable patients with FN. Several studies in the literature have demonstrated that CISNE provides superior discriminatory power compared with the MASCC score in identifying high-risk patients. These findings are consistent with our results, in which intermediate and high CISNE risk categories emerged as independent predictors of prolonged hospital stay [12,13].

This study has several limitations. Its single-center and retrospective design restricts the generalizability of the findings, and the inclusion of only solid-tumor patients limits applicability to hematologic malignancies. The absence of MASCC scoring prevented direct comparison with widely used prognostic indices. As no inpatient mortality occurred and long-term survival data were unavailable, length of hospital stay was used as an indirect clinical outcome. The small number of Intensive Care Unit (ICU) transfers and limited sample sizes in certain tumor subtypes hindered subgroup and tumor-specific analyses, while incomplete retrospective documentation of TNM staging restricted staging evaluation to metastasis status. Moreover, hospitalization duration may be affected by non-medical factors, such as discharge delays or social constraints, introducing potential bias. In addition, several clinically relevant infection-related variables—including time-to-antibiotics, clearly documented infection subtypes (e.g., pneumonia, urinary tract infection, or catheter-related infection), initial vital signs or sepsis-related parameters, and the adequacy of empiric antimicrobial coverage—could not be consistently retrieved. Although culture positivity was analyzed, detailed information on pathogen distribution and infection subtype classification was not uniformly available, limiting infection-source–specific assessment. Furthermore, detailed data regarding chemotherapy regimens and cycle numbers were not consistently accessible, which may have restricted adjustment for treatment-related factors. Future prospective, multicenter studies with larger cohorts are needed to validate these findings.

## 5. Conclusions 

In this study, a low HALP score, intermediate and high CISNE risk categories, and microbiological culture positivity were identified as independent predictors of prolonged hospitalization among patients with solid tumors who developed FN. These findings highlight the potential utility of the HALP score—an easily obtainable and cost-effective immunonutritional biomarker—in early clinical risk stratification. Although the PNI score showed significance in univariate analysis, it did not retain its predictive value after adjustment for stronger clinical variables, and the CAR demonstrated no meaningful association with hospitalization duration.

The incorporation of HALP into FN assessment algorithms, alongside validated prognostic systems such as MASCC and CISNE, may enhance early identification of patients at higher risk for unfavorable clinical trajectories, including prolonged hospitalization. Patients presenting with markedly low HALP values may warrant closer observation at admission, more cautious monitoring during the early phases of evaluation, or prioritization for timely supportive interventions depending on the overall clinical context. Importantly, these potential applications remain exploratory, and prospective multicenter studies are required to determine whether integrating HALP into existing FN risk models can improve predictive accuracy and meaningfully inform triage decisions and clinical management strategies.

## Figures and Tables

**Table 1:** Clinical and Demographic Characteristics of the Patients.

Characteristic	Value
Number of patients	169
Age (years), median [min–max]	64 [19–81]
<65	82 (51.5)
≥65	87 (48.5)
Sex	
Female, *n* (%)	86 (50.9)
Male, *n* (%)	83 (49.1)
Stage	
Metastatic, *n* (%)	87 (51.8)
Non-metastatic, *n* (%)	81 (48.2)
Comorbidity	
Present, *n* (%)	86 (50.9)
Absent, *n* (%)	83 (49.1)
Positive microbiological culture	
Present, *n* (%)	57 (33.7)
Absent, *n* (%)	112 (66.3)
Primary cancer type	
Lung	53 (31.4)
Head and neck	27 (16.0)
Breast	24 (14.2)
Gastric	16 (9.4)
Ovarian–Endometrial	16 (9.4)
Pancreatic	12 (7.1)
Colon	12 (7.1)
Rectal	4 (2.4)
Sarcoma	3 (1.8)
Others	2 (1.2)
CISNE Category	
Low risk	51 (30.2)
Intermediate risk	67 (39.6)
High risk	51 (30.2)
Length of hospital stay (days), median [min–max]	9 [3–29]

Abbreviations: CISNE, Clinical Index of Stable Febrile Neutropenia.

**Table 2:** Laboratory Parameters and Calculated Scores.

Parameter	Median	Minimum	Maximum
Hemoglobin (g/dL)	9.6	5.8	11.8
Albumin (g/L)	32	13	51
Total protein (g/L)	55	38	73
Neutrophil count (/µL)	120	10	940
Lymphocyte count (/µL)	370	10	1860
Monocyte Count (/µL)	250	40	1230
C-reactive protein (CRP, mg/L)	99	18	402
Platelet count (/µL)	84,000	2000	326,000
HALP score	17.46	0.8	276
CAR score	3.33	0.62	22.22
PNI score	35.50	13.35	60.30

Abbreviations: HALP, Hemoglobin–Albumin–Lymphocyte–Platelet; CAR, C-reactive protein/Albumin ratio; PNI, Prognostic Nutritional Index.

**Table 3:** Summary of microbiological culture results in patients with febrile neutropenia.

Culture type	*n* (%)
Any positive microbiological culture	57 (33.7)
Urine culture positive	20 (35.1)
Blood culture positive	15 (26.3)
Respiratory culture positive	17 (29.8)
Stool culture positive	10 (17.5)
Multiple culture sites positive	12 (21.1)

Footnote: Blood cultures were routinely obtained from all patients at admission, whereas urine, respiratory, and stool cultures were requested selectively based on the clinical presentation. Percentages may exceed 100% because some patients had positivity at more than one culture site.

**Table 4:** Spearman Correlation Analysis (with Length of Hospital Stay).

Variable	r	*p*
Neutrophil count (/µL)	0.004	0.955
Albumin (g/L)	−0.184	0.017
C-reactive protein (CRP, mg/L)	−0.014	0.858
Age	0.002	0.982
HALP	−0.469	<0.001
CAR	−0.039	0.617
PNI	−0.273	<0.001

Abbreviations: HALP, Hemoglobin–Albumin–Lymphocyte–Platelet; CAR, C-reactive protein/Albumin ratio; PNI, Prognostic Nutritional Index. Spearman’s rho test (two-tailed); *p* < 0.05 was considered statistically significant.

**Table 5:** Univariate and Multivariate Logistic Regression Analysis of Factors Associated With Prolonged Hospital Stay.

Variable	Univariate OR (95% CI)	*p*-Value	Multivariate OR (95% CI)	*p*-Value
Sex (Male vs. Female)	0.464 (0.249–0.863)	0.015	0.468 (0.156–1.406)	0.176
Positive microbiological culture (Yes vs. No)	8.395 (4.030–17.489)	<0.001	11.279 (3.595–35.387)	<0.001
CISNE Intermediate vs. Low risk	4.756 (1.779–12.715)	0.002	8.620 (1.807–41.121)	0.007
CISNE High risk vs. Low Risk	30.750 (10.266–92.103)	<0.001	67.490 (12.024–378.816)	<0.001
HALP (Low vs. High)	28.57 (8.23–99.00)	<0.001	47.62 (9.26–238.0)	<0.001
PNI (Low vs. High)	3.16 (1.59–6.25)	0.001	1.64 (0.52–5.21)	0.400
Age (High vs. Low)	0.582 (0.315–1.075)	0.084	-	-
Stage (Metastatic vs. Non-metastatic)	1.661 (0.897–3.077)	0.107	-	-
Comorbidity (present vs. absent)	1.861 (0.897–3.077)	0.551	-	-

Abbreviations: HALP, Hemoglobin–Albumin–Lymphocyte–Platelet; PNI, Prognostic Nutritional Index; CISNE, Clinical Index of Stable Febrile Neutropenia. In the multicollinearity analysis, all variables demonstrated VIF values below 2.5, indicating that no meaningful multicollinearity was present in the model.

## Data Availability

Data supporting the reported results are available from the corresponding author upon reasonable request.

## References

[1] Cossey J., Cote M.C.B. (2024). Evaluation and management of febrile neutropenia in patients with cancer. JAAPA.

[2] Seltzer J.A., Frankfurt O., Kyriacou D.N. (2022). Association of an emergency department febrile neutropenia intervention protocol with time to initial antibiotic treatment. Ann. Emerg. Med..

[3] Stephens R.S., Nates J.L., Price K.J. (2019). Neutropenic fever in the intensive care unit. Oncologic Critical Care.

[4] Gould Rothberg B.E., Quest T.E., Yeung S.C.J., Pelosof L.C., Gerber D.E., Seltzer J.A., Bischof J.J., Thomas C.R., Akhter N., Mamtani M. (2022). Oncologic emergencies and urgencies: A comprehensive review. CA Cancer J. Clin..

[5] Boccia R., Glaspy J., Crawford J., Aapro M. (2022). Chemotherapy-induced neutropenia and febrile neutropenia in the US: A beast of burden that needs to be tamed?. Oncologist.

[6] Farag C.M., Antar R., Akosman S., Ng M., Whalen M. (2023). What is hemoglobin, albumin, lymphocyte, platelet (HALP) score? A comprehensive literature review of HALP’s prognostic ability in different cancer types. Oncotarget.

[7] Wu M., Guo J., Guo L., Zuo Q. (2016). The C-reactive protein/albumin ratio predicts overall survival of patients with advanced pancreatic cancer. Tumour Biol..

[8] Pinato D.J., North B.V., Sharma R. (2012). A novel, externally validated inflammation-based prognostic algorithm in hepatocellular carcinoma: The prognostic nutritional index (PNI). Br. J. Cancer.

[9] Wang X., He Q., Liang H., Liu J., Xu X., Jiang K., Zhang J. (2021). A novel robust nomogram based on preoperative hemoglobin and albumin levels and lymphocyte and platelet counts (HALP) for predicting lymph node metastasis of gastric cancer. J. Gastrointest. Oncol..

[10] Xu H., Zheng X., Ai J., Yang L. (2023). Hemoglobin, albumin, lymphocyte, and platelet (HALP) score and cancer prognosis: A systematic review and meta-analysis of 13,110 patients. Int. Immunopharmacol..

[11] Li M.X., Liu X.M., Zhang X.F., Zhang J.F., Wang W.L., Zhu Y., Dong J., Cheng J.W., Liu Z.W., Ma L. (2014). Prognostic role of neutrophil-to-lymphocyte ratio in colorectal cancer: A systematic review and meta-analysis. Int. J. Cancer.

[12] Coyne C.J., Le V., Brennan J.J., Castillo E.M., Shatsky R.A., Ferran K., Brodine S., Vilke G.M. (2017). Application of the MASCC and CISNE Risk-Stratification Scores to Identify Low-Risk Febrile Neutropenic Patients in the Emergency Department. Ann. Emerg. Med..

[13] Ahn S., Rice T.W., Yeung S.J., Cooksley T. (2018). Comparison of the MASCC and CISNE scores for identifying low-risk neutropenic fever patients: Analysis of data from three emergency departments of cancer centers in three continents. Support. Care Cancer.

[14] Chindaprasirt J., Wanitpongpun C., Limpawattana P., Thepsuthammarat K., Sripakdee W., Wirasorn K., Sookprasert A. (2013). Mortality, length of stay, and cost associated with hospitalized adult cancer patients with febrile neutropenia. Asian Pac. J. Cancer Prev..

[15] Bachlitzanaki M., Aletras G., Bachlitzanaki E., Messaritakis I., Koukias S., Koulouridi A., Bachlitzanakis E., Kaloeidi E., Vakonaki E., Kontopodis E. (2023). Evaluation of febrile neutropenia in hospitalized patients with neoplasia undergoing chemotherapy. Medicina.

[16] Rosa R.G., Goldani L.Z. (2014). Factors associated with hospital length of stay among cancer patients with febrile neutropenia. PLoS ONE.

[17] Pathak R., Giri S., Aryal M.R., Karmacharya P., Bhatt V.R., Martin M.G. (2015). Mortality, length of stay, and health care costs of febrile neutropenia-related hospitalizations among patients with breast cancer in the United States. Support. Care Cancer.

[18] Özlüer Y.E., Güvenç F.D. (2022). Does the waiting time for admission in the emergency department increase mortality in febrile neutropenia patients? A retrospective observational study. Anatol. J. Emerg. Med..

[19] Dimitrijević J., Čalamać M., Đurmez O., Krstić D., Stojanović M. (2024). Serum albumin as a prognostic biomarker for febrile neutropenia outcome and complications: A prospective observational trial. Clin. Med. Insights Oncol..

[20] Dimitrijević J., Čalamać M., Đurmez O., Stojanović M. (2025). Mean platelet volume-to-albumin ratio as a predictor of mortality in patients with febrile neutropenia: An observational study. Medicina.

[21] Inagaki T., Sato M., Kin M., Otsuka K., Murakami M., Kogo M. (2022). Predictive factors of febrile neutropenia after primary prophylactic pegfilgrastim with DCF chemotherapy for esophageal cancer. Anticancer Res..

